# A Conversation with Waldemar A. Carlo, MD, Co-Division Director of Neonatology, Edwin M. Dixon Chair in Neonatology Professor of Pediatrics, University of Alabama at Birmingham

**DOI:** 10.1017/cts.2024.602

**Published:** 2024-10-24

**Authors:** Clinical Research Forum

**Affiliations:** Clinical Research Forum, Washington, DC, USA

## Top 10 Clinical Research Achievement Awards Q & A

This article is part of a series of interviews with recipients of Clinical Research Forum’s Top 10 Clinical Research Achievement Awards. This interview is with Waldemar A. Carlo, MD, Co-Division Director of Neonatology, Edwin M. Dixon Chair in Neonatology, Professor of Pediatrics, University of Alabama at Birmingham. Dr Carlo’s research is focused on reducing mortality and major morbidities during early childhood in the USA and developing countries. He received a 2024 Top 10 Clinical Research Achievement Award for **Azithromycin to Prevent Sepsis or Death in Women Planning a Vaginal Birth**. *The interview has been edited for length and clarity.*

## When did you first become interested in a career in medicine?

I have always loved science and biology, and I have always loved working with people. So when I was young, I started to think about how I could combine these interests. Also, my parents always encouraged academics. My dad was an engineer and my mom was an assistant to the chancellor of a university. Going to college and then to medical school was a good path for me.

## Why did you decide to specialize in neonatology?

When I was in my third year of medical school, I found that the rotations in pediatrics were the best as I felt I could make the biggest impact – and then, the very best of those were the rotations in newborn intensive care. I could see that neonatology is one of the specialties that helps you do the most good. You have a direct impact on the patient and their loved ones, their family.

## What motivated you to pursue neonatal and childhood research?

During my training in newborn care, I realized that in order to do the most good for babies, I would have to do research. This was back in the 1980s, and there were so many unanswered questions. Since then, major advances have been made. For example, when I was training, most babies in the USA weighing below 2 lbs. or so would not survive. Now, those babies have a very good chance of survival. But still, there is a lot that is unknown. That’s why for many years now I have been involved in neonatal and childhood research, not just in the USA, but in developing countries, too. This includes single-center trials and multicenter trials such as the First Breath Trial, the BRAIN-HIT Trial, and the SUPPORT Trial. I’m focused on trials to improve perinatal care, which includes the baby and the mother at the time she’s going to deliver.

## That’s a good segue to your award-winning research. What was the genesis of that clinical trial?

Maternal infections, particularly sepsis, during the peripartum period account for 10% of maternal deaths and are among the top three causes of maternal death worldwide. Previous research by my colleague Dr Alan Tita showed that adjunctive intravenous azithromycin prophylaxis performed during labor can lower this risk for cesarean delivery. But no large-scale research had been done for vaginal delivery. With maternal infections such a common problem for women, we needed to ask that question. Our trial was the first large-scale study conducted in lower- and middle-income countries to test whether a single oral dose of azithromycin in women in labor who were planning a vaginal delivery would reduce maternal sepsis or death along with stillbirth or neonatal death or sepsis.

## How do you coordinate such a large multicenter trial?

It’s a huge collaborative effort. Our team members screened over 44,000 pregnant women across eight sites in the Global Network for Women’s and Children’s Health Research. Ultimately, more than 29,000 women underwent randomization. A large-scale trial like this can only happen with the leadership and participation of local clinicians and research staff. They train in research protocols, data collection, and the basics of patient assessment. We had to have local personnel who could conduct the study – that’s the only way to sustain the capacity that we built. All of this is made possible by the National Institute of Child Health and Human Development. We work with partner universities and other institutes in low-resource settings. It takes a lot of coordination, but it’s worth it because the research is so important.

## What were the results of the trial?

That data showed that among women planning a vaginal delivery, a single oral dose of azithromycin resulted in a significantly lower risk of maternal sepsis or death than placebo. That means we have a simple oral, low-cost treatment to potentially have a significant impact on moms; however, the data showed little effect on stillbirth or neonatal sepsis or death, and additional long-term studies are needed to further inform the reproducibility and generalizability of our findings.

## How do you balance your clinical practice with your research? Do you ever think about choosing just one?

Choosing just one – clinical practice or research – would be the easy path. Sometimes, one must take the important path. When you see patients, you see first-hand how an infection can impact the way a mother is able to care for her baby. You see that this is a very important issue and then you ask, how can we do better? I feel the responsibility of not going along with the status quo but to try to do better. That’s why we do the research – so we can provide better care for moms and their babies. It always starts with taking care of a patient and looking for ways to do better.

## What do you enjoy when you’re not working and how does it impact your work?

I am a family man, so I love to be with my wife, our four adult children, and 12 grandkids. For the holiday coming up, I’m going to be babysitting for the whole weekend, and I’m so happy about that – I love being with all of them. I’m relentless in trying to do things the best way possible at home and at work. When I was young, I was a Boy Scout, and they always promise to do the best they can. I’ve spent my life trying to live up to that and will continue to do so. Like Robert Frost said, “I have promises to keep and miles to go before I sleep.”

